# CRISPR-driven strategies to disrupt methicillin-resistant *Staphylococcus aureus* biofilms: a review

**DOI:** 10.3389/fcimb.2026.1784529

**Published:** 2026-05-13

**Authors:** Jose Jurel M. Nuevo, Jamil Allen G. Fortaleza, Kevin Smith P. Cabuhat, Christian Joseph N. Ong, Anna C. Jalova, Ferdinand A. Mortel, Grace D. Bacalzo

**Affiliations:** 1 College of Medical Laboratory Science, Our Lady of Fatima University, Valenzuela City, Philippines; 2 National University Philippines, Manila, Philippines; 3 Department of Biology, College of Science, De La Salle University, Manila, Philippines; 4 Basic Education Department, La Consolacion University Philippines, Malolos, Bulacan, Philippines; 5 College of Medical Technology, Manila Central University, Caloocan City, Philippines; 6 College of Medical Technology, Centro Escolar University, Malolos, Philippines

**Keywords:** antibiotic resistance, antimicrobials, efflux pump, quorum sensing, β-lactam

## Abstract

Methicillin-resistant *Staphylococcus aureus* exhibits heightened tolerance to antimicrobial therapy through restricted drug penetration, extracellular polymeric substance-mediated protection, metabolic heterogeneity, and persister cell formation, limiting the effectiveness of current treatment strategies. CRISPR-Cas systems have emerged as programmable antimicrobial tools capable of targeting resistance genes, virulence determinants, and regulatory pathways; however, existing approaches remain largely gene-centric and insufficiently integrated with the biological complexity of biofilm-associated infections. This review aims to provide a comprehensive and integrative analysis of CRISPR-based strategies for targeting MRSA biofilms by linking molecular CRISPR mechanisms with key biofilm processes and evaluating their translational potential. CRISPR-Cas systems have emerged as programmable antimicrobial platforms with the ability to selectively target resistance genes, virulence factors, and regulatory networks. In MRSA biofilms, these systems are increasingly being explored for their potential to disrupt biofilm-associated determinants and weaken the molecular basis of persistence. Recent advances involving Cas9, Cas12a, and Cas13 highlight the potential of CRISPR-based targeting to interfere with resistance mechanisms, quorum sensing pathways, and structural components relevant to biofilm stability. Emerging *in vivo* studies, particularly those using engineered bacteriophages and localized delivery systems, provide early evidence that CRISPR-based strategies can reduce bacterial burden and impair biofilm integrity under physiologically relevant conditions. Nevertheless, significant barriers remain, including limited penetration into mature biofilms, delivery inefficiency, off-target activity, immunogenicity, resistance evolution, and regulatory uncertainty. Ultimately, CRISPR-based interventions represent a promising but still developing approach for the control of MRSA biofilm-associated infections, requiring further refinement in delivery design, target selection, and translational validation.

## 1 Introduction

While existing reviews have extensively explored CRISPR technologies as antimicrobial tools, particularly in targeting antimicrobial resistance (AMR) through gene editing and plasmid curing (Fagen et al., 2017; Ali Agha et al., 2025; de Souza et al., 2025; Barrangou and van Pijkeren, 2016; Ghosh et al., 2022), these approaches are frequently framed within a gene-centric perspective that inadequately reflects the biological complexity of real-world infections. This limitation is especially evident in biofilm-associated diseases, where resistance is not solely dictated by genetic determinants but is shaped by dynamic physiological states, structured microbial communities, and tightly regulated adaptive responses. Although prior studies have acknowledged the potential of CRISPR in biofilm inhibition, mechanistic integration remains limited, particularly in how CRISPR systems interact with key biofilm processes such as quorum sensing (QS) regulation, extracellular polymeric substance (EPS) production, and biofilm-specific resistance phenotypes (Sharma et al., 2022; Tao et al., 2022; Dutta et al., 2023). Consequently, current frameworks often fail to connect molecular targeting with the ecological and spatial complexity of biofilms, constraining the translational applicability of CRISPR-based strategies.

This gap is particularly critical in the context of methicillin-resistant *Staphylococcus aureus* (MRSA), a globally significant pathogen characterized by the convergence of multidrug resistance and biofilm-mediated persistence (Guo et al., 2020). Despite advances in antimicrobial therapy and infection control, MRSA continues to impose substantial morbidity and mortality worldwide, with *S. aureus* bacteremia exhibiting 30-day mortality rates of 20–40% even with appropriate treatment (Melzer and Welch, 2013). MRSA remains endemic across healthcare systems, affecting over 150,000 patients annually in the European Union and contributing to a significant number of infections and deaths in the United States (Köck et al., 2010; Xu et al., 2025). A major contributor to this burden is its capacity to form biofilms on medical devices and host tissues, leading to persistent infections that are refractory to both antimicrobial therapy and immune clearance (Simões et al., 2009; Ćirković et al., 2015). Current therapeutic approaches, although widely implemented, are often limited by poor biofilm penetration, emerging resistance, and high recurrence rates. These strategies and their associated limitations are summarized in **Table 1**.

**Table 1:** Current MRSA management strategies and their limitations.

Strategy	Examples	Mechanism of action	Clinical use	Key limitations	References
β-lactam Alternatives	Vancomycin, Daptomycin, Linezolid, Ceftaroline	Inhibit cell wall synthesis; membrane disruption; protein synthesis inhibition	First-line and second-line MRSA treatment	Poor biofilm penetration; reduced activity in dormant cells; emerging resistance; toxicity	(Lade and Kim, 2021; Bi et al., 2022)
Combination Antibiotic Therapy	Vancomycin + Rifampicin; Daptomycin + β-lactams	Synergistic multi-target antimicrobial action	Severe or persistent infections	Variable efficacy; increased toxicity; does not fully eradicate biofilms	(Antony, 2006)
Anti-biofilm Agents	DNase, Dispersin B, EDTA	Degradation of EPS components	Adjunct therapy	Temporary disruption; biofilm regrowth; limited clinical validation	(Shrestha et al., 2022)
Antimicrobial Peptides (AMPs)	LL-37, Synthetic peptides	Membrane disruption; biofilm inhibition	Experimental	Instability; cytotoxicity; high cost	(Cabuhat et al., 2025)
Phage Therapy	Lytic bacteriophages	Bacterial lysis via infection	Clinical trials / compassionate use	Narrow host range; resistance; regulatory barriers	(Fortaleza et al., 2024)
Nanoparticle-Based Therapies	Silver nanoparticles, Liposomes	Enhanced drug delivery and penetration	Experimental / adjunct	Cytotoxicity; delivery inconsistency; scalability issues	(Ong et al., 2025)
Quorum Sensing Inhibitors (QSIs)	RNAIII-inhibiting peptides (RIPs)	Disruption of virulence signaling	Experimental	Limited standalone efficacy; pathway redundancy	(Hetta et al., 2024)

In this context, CRISPR (Clustered Regularly Interspaced Short Palindromic Repeats) technologies represent a paradigm shift from nonspecific antimicrobial activity toward programmable (Karimi et al., 2025), sequence-specific intervention (Huang et al., 2022). Unlike traditional antibiotics, CRISPR–Cas systems enable precise targeting of resistance genes, virulence determinants, and regulatory pathways, offering the potential to disrupt the molecular drivers of persistence. Early applications in MRSA have demonstrated the ability to target resistance determinants such as *mecA*, restoring β-lactam susceptibility and enhancing the efficacy of conventional antibiotics (Hernández-Cuellar et al., 2023; Hosseini et al., 2020; Li et al., 2016; Fishovitz et al., 2014; Hiramatsu et al., 1997). However, these strategies remain limited by their focus on single-gene targets and insufficient consideration of biofilm-associated complexity. Biofilm architecture, governed by multilayered regulatory networks and protected by a dense EPS matrix, restricts CRISPR delivery and reduces genome-editing efficiency, particularly within mature biofilms (Reed et al., 2024; Zhu et al., 2025; Aboelnaga et al., 2024; Alallam et al., 2021; Bhuiyan, 2025; Zuberi et al., 2024). These constraints highlight the need for integrative approaches that align CRISPR system design with biofilm physiology and delivery strategies to achieve clinically meaningful outcomes.

The objective of this review is to provide a mechanistic and translational synthesis of CRISPR-based strategies for disrupting MRSA biofilms, with a particular focus on integrating gene-targeting approaches with biofilm physiology, delivery systems, and clinical applicability.

## 2 Biology of MRSA biofilm formation

Initial attachment represents a highly regulated phase in which MRSA integrates environmental cues to commit to surface colonization rather than a passive adhesion event. This process is mediated by microbial surface components recognizing adhesive matrix molecules (MSCRAMMs), particularly ClfA and ClfB, which facilitate specific interactions with host extracellular matrix proteins and support both tissue tropism and device-associated colonization (Aboelnaga et al., 2024; Vlaeminck et al., 2022; Patel and Rawat, 2023; Niek et al., 2021; Haddad et al., 2018). Adhesin expression is dynamically modulated by environmental conditions, including subinhibitory antibiotic exposure, suggesting that early antimicrobial intervention may unintentionally promote biofilm initiation (Kaushik et al., 2024). The transition to microcolony formation reflects a shift toward coordinated population-level behavior, driven by the expression of genes such as *icaADBC*, *fnbA*, *fnbB*, and *atl* (Patel and Rawat, 2023; Kaushik et al., 2024; Mirzaee et al., 2014). This transition is regulated by the *agr* quorum-sensing system, emphasizing that biofilm development is governed by density-dependent signaling networks. Variability in these regulatory pathways among MRSA strains contributes to heterogeneity in biofilm formation, complicating the development of broadly effective therapeutic strategies (Shivaee et al., 2019).

As biofilms mature, MRSA establishes a structurally complex EPS matrix that functions as both a physical scaffold and a protective barrier. The integration of polysaccharide intercellular adhesin (PIA), extracellular DNA (eDNA), wall teichoic acids, and capsular polysaccharides stabilizes the biofilm and modulates interactions with antimicrobial agents and host defenses (Patel and Rawat, 2023; Hajiagha and Kafil, 2023). Matrix development is actively regulated, with controlled autolysis via the *cidABC* operon contributing to eDNA release and structural cohesion (Aboelnaga et al., 2024). Concurrently, metabolic adaptation under nutrient-limited and hypoxic conditions generates physiologically distinct subpopulations with varying susceptibility to antimicrobial agents (Vlaeminck et al., 2022). This spatial and metabolic heterogeneity creates microenvironments that limit antimicrobial penetration and efficacy, representing a central barrier to treatment.

The dispersal phase reflects a regulated transition that enables MRSA to exit the biofilm and establish new infection sites. Proteolytic degradation of the EPS matrix, combined with *agr*-mediated upregulation of virulence factors, facilitates the release of cells capable of colonization and dissemination (Aboelnaga et al., 2024; Patel and Rawat, 2023). Environmental stressors, including antibiotic exposure and nutrient depletion, can actively trigger this process (Aboelnaga et al., 2024; Kaushik et al., 2024). Clinically, dispersal is associated with bacteremia and metastatic infection, reinforcing the role of biofilms as reservoirs of persistent and systemic disease (Kaushik et al., 2024; Sato et al., 2019). Overall, MRSA biofilm formation is governed by an interplay between structural development, metabolic adaptation, and regulatory control, resulting in a heterogeneous system that challenges conventional antimicrobial approaches and necessitates targeted, mechanism-driven interventions (Paulander et al., 2018; Sionov and Steinberg, 2022; Peng et al., 2022; Durham et al., 2021; Tang et al., 2023; Wang et al., 2025; Jolivet-Gougeon and Bonnaure-Mallet, 2014).

## 3 Biofilm-associated resistance mechanisms in MRSA

MRSA represents a major global clinical threat, largely driven by its capacity to form biofilms that enhance persistence and therapeutic failure (Usun Jones et al., 2022; Hegazy et al., 2025; Donlan and Costerton, 2022). A substantial proportion of clinical isolates, estimated at 76–90%, exhibit biofilm-forming capability, frequently in association with multidrug resistance, reinforcing MRSA’s classification as a high-priority antimicrobial resistance pathogen (Usun Jones et al., 2022; Hegazy et al., 2025; Hibbitts and O’Leary, 2018). In clinical settings, biofilm-associated MRSA is strongly linked to device-related infections and invasive diseases such as infective endocarditis, contributing to prolonged hospitalization and recurrent infection (Kaushik et al., 2024; Luther et al., 2018; Wang et al., 2025). These outcomes reflect a convergence of structural, physiological, and genetic mechanisms that collectively promote survival under antimicrobial pressure, underscoring that MRSA resistance extends beyond classical genetic determinants. In addition to its global burden, MRSA is characterized by substantial genetic and clonal diversity, which influences virulence, resistance profiles, and epidemiological distribution. These major MRSA lineages and their associated characteristics are summarized in **Table 2**.

**Table 2:** Genetic diversity, clonal lineages, and epidemiological characteristics of MRSA.

Clonal complex (CC)	Sequence type (ST)	SCCmec type(s)	Key features	Epidemiological and clinical significance	References
CC5	ST5	I, II, IV	Hospital-adapted lineage; multidrug resistance	Common in healthcare-associated MRSA (HA-MRSA); associated with nosocomial outbreaks	Fortaleza et al., 2024; Ong et al., 2025; Bae et al., 2006; Chambers and Deleo, 2009
CC8	ST239, ST8	III, IV	Includes Brazilian epidemic clone (ST239) and USA300 (ST8); high adaptability	ST239: multidrug-resistant hospital lineage; ST8 (USA300): highly virulent community-associated strain	Melzer and Welch, 2013; Neyra et al., 2015; Fishovitz et al., 2014
CC22	ST22	IV	Contains epidemic MRSA-15 (EMRSA-15); high transmissibility	Dominant hospital-associated lineage in Europe and Middle East	Köck et al., 2010
CC30	ST30	IV	Often PVL-positive; associated with increased virulence	Community-associated MRSA; prevalent in South America and other regions	Xu et al., 2025; Boyle-Vavra and Daum, 2007
CC59	ST59	IV	Genetically diverse lineage; moderate resistance	Common community-associated MRSA in Asia	Davies, 2003
CC398	ST398	IV, V, VII	Livestock-associated MRSA (LA-MRSA); zoonotic transmission	Emerging global public health concern; occupational exposure risk	Donlan and Costerton, 2002; Simões et al., 2009

A central mechanism underlying this tolerance is the restricted penetration of antibiotics through the EPS matrix. This matrix forms a dense, three-dimensional scaffold composed of polysaccharides, proteins, eDNA, lipids, and teichoic acids, which collectively limit diffusion of antimicrobial agents and generate sub-inhibitory concentration gradients within the biofilm (Pan et al., 2016; Vu et al., 2009; Hasan and Aggarwal, 2025). Matrix production is tightly regulated by genes such as *icaA* and *icaD* (Armoon et al., 2024), alongside adhesins (*fnbA*, *fnbB*, *clfA*, *clfB*) and regulatory systems including *atl*, *arlS*, *cidC*, and *cidR* (Gangwar et al., 2020; Haddad et al., 2018; Shivaee et al., 2019; Cruz Martínez et al., 2016; Lin et al., 2017; Cramton et al., 1999). Controlled autolysis further contributes to eDNA release, enhancing matrix cohesion and antibiotic sequestration (Peng et al., 2022). Clinically, this barrier significantly reduces the efficacy of antibiotics such as β-lactams and glycopeptides, whereas agents like aminoglycosides and fluoroquinolones demonstrate comparatively improved penetration (Kaushik et al., 2024; Mirzaee et al., 2014; Hamady et al., 2024). The restoration of antibiotic susceptibility following enzymatic degradation of EPS components further supports the matrix as a critical therapeutic target (Rao et al., 2025).

Beyond structural protection, MRSA biofilms harbor persister cells, phenotypically dormant subpopulations capable of surviving lethal antibiotic exposure without genetic resistance (Karkhur et al., 2025; Wuyts et al., 2018; Cordeiro et al., 2021; Mohan et al., 2023). These cells arise through activation of stress-response pathways, including stringent response signaling (RelA/SpoT), toxin–antitoxin systems, and SOS-mediated responses (Karkhur et al., 2025; Cordeiro et al., 2021; Mohan et al., 2023; Narimisa et al., 2020). The biofilm environment further enhances persister survival by limiting both antibiotic exposure and immune clearance (Karkhur et al., 2025; Wuyts et al., 2018; Cordeiro et al., 2021). Clinically, persister-enriched biofilms are strongly associated with chronic and relapsing infections, including cystic fibrosis–associated lung infections and implant-related infections, highlighting a key limitation of antibiotics that primarily target metabolically active cells (Lu et al., 2023; Singh et al., 2023; Butini et al., 2019).

In parallel, MRSA biofilms undergo extensive metabolic reprogramming that enables survival under nutrient limitation and environmental stress (Gambino and Cappitelli, 2016). Biofilm-associated cells exhibit selective activation of metabolic pathways, including upregulation of tricarboxylic acid (TCA) cycle genes such as *fumC*, which contributes to enhanced biofilm production and matrix biosynthesis (Gabryszewski et al., 2019; Ammons et al., 2014). Metabolic intermediates further regulate biofilm dynamics through post-translational modifications, including lysine succinylation, linking cellular metabolism to virulence regulation (Zhou et al., 2026; Zhu et al., 2025). Compared with methicillin-sensitive strains, MRSA biofilms often display reduced metabolic activity, contributing to increased antibiotic tolerance (Li et al., 2025). Additionally, stress-adaptive responses, including resistance to oxidative stress, acidity, heat, and osmotic pressure, reinforce biofilm resilience (Ma et al., 2025). Enhanced antioxidant defenses, such as increased staphyloxanthin production and upregulation of *sodA* and *katA*, protect cells from reactive oxygen species (Yu et al., 2024), while small colony variants (SCVs) further contribute to persistence through metabolic dormancy and tolerance (Mirani et al., 2015, Ali Mirani et al., 2018).

Finally, MRSA biofilms serve as hubs for horizontal gene transfer (HGT), accelerating the dissemination of antibiotic resistance and virulence determinants. The high cell density and spatial organization within biofilms enhance transformation, conjugation, and transduction processes (Bourassa et al., 2022; Tran et al., 2023; Diep et al., 2006; Craft et al., 2019). Natural transformation enables acquisition of SCCmec elements via the Ccr-attB system (Juhas, 2015), while conjugative transfer is facilitated by close cellular proximity (Tran et al., 2023; Diep et al., 2006). Sub-inhibitory antibiotic exposure can further stimulate phage-mediated transduction, promoting genetic exchange and adaptation (Maree et al., 2024; Ma et al., 2021). These processes contribute to the emergence of epidemic lineages such as USA300 and highlight the role of biofilms in MRSA evolution (Bourassa et al., 2022; Lécuyer et al., 2018; Shokrollahi et al., 2022; Stanczak-Mrozek et al., 2017). Underpinning these mechanisms are global regulators such as *sarA* and *agr*, which coordinate biofilm stability, virulence expression, and resistance phenotypes, further emphasizing the complexity of MRSA biofilm-associated persistence (Li et al., 2016; Kuai et al., 2024; Abdelhady et al., 2014; Kim et al., 2022; Rajasree et al., 2016; Da et al., 2017; Nielsen et al., 2014; Sun et al., 2012; Bernabè et al., 2021; Yamaguchi et al., 2024; Bibalan et al., 2014).

## 4 New perspectives and advances in the use of CRISPR for MRSA biofilms

Recent advances in CRISPR technology have shifted its role from a molecular editing tool toward a translational platform capable of addressing the persistent challenge of MRSA biofilms. Emerging studies increasingly emphasize combinatorial and systems-level approaches, particularly multiplex editing strategies that simultaneously target resistance determinants and biofilm-regulatory networks, thereby overcoming pathway redundancy that limits single-gene interventions (Ates et al., 2024; Mayorga-Ramos et al., 2023). In parallel, CRISPR-based modulation of quorum sensing and regulatory circuits has demonstrated the ability to disrupt coordinated bacterial behavior, interfering with biofilm stability beyond direct gene disruption (Saffari-Natanzi et al., 2025; Ong et al., 2025; Pandey and Vavilala, 2024). These developments highlight a shift toward more integrated strategies that address the complexity of biofilm-associated infections.

A major area of innovation lies in the development of delivery systems tailored to the biofilm microenvironment. Nanoparticle-based platforms, including liposomal and polymeric carriers, enhance CRISPR stability, facilitate penetration through the EPS matrix, and enable controlled release, resulting in significant reductions in biofilm biomass (Saffari-Natanzi et al., 2025; Yoon et al., 2025). Complementary approaches such as phagemids and conjugative plasmids exploit natural bacterial processes for targeted and potentially self-propagating delivery (Mayorga-Ramos et al., 2023; Yoon et al., 2025). Increasingly, CRISPR is being integrated into multimodal antimicrobial strategies, combining with nanoparticles, antimicrobial peptides, bacteriophages, and micro/nanomotor systems to enhance efficacy through simultaneous structural and molecular disruption (Joshi et al., 2025; Cabuhat et al., 2025; Sun et al., 2026). Despite these advances, clinical translation remains constrained by challenges in delivery efficiency, off-target effects, biosafety, and broader regulatory and ecological considerations (Saffari-Natanzi et al., 2025; Yoon et al., 2025; Gager and Flores-Mireles, 2024).

## 5 Mechanisms of action of Cas9, Cas12a, and Cas13 in MRSA biofilm disruption

CRISPR-Cas9 functions through a single-guide RNA (sgRNA) that directs the nuclease to specific DNA sequences, inducing double-stranded breaks (DSBs) that are repaired via non-homologous end joining (NHEJ) or homology-directed repair (HDR) (Banerjee et al., 2024; De La Fuente-Núñez and Lu, 2017; Ebrahimi-Khezrabad et al., 2025). In MRSA, this allows targeted disruption of key biofilm-associated genes such as the *ica* operon, which mediates polysaccharide intercellular adhesin (PIA) production (Cui et al., 2015), and the quorum-sensing regulator *agr*. These interventions impair biofilm structural integrity, reduce intercellular communication, and attenuate virulence, thereby weakening bacterial persistence under antimicrobial pressure (De La Fuente-Núñez and Lu, 2017; Ong et al., 2025). Importantly, Cas9 enables stable and heritable genomic modifications, making it suitable for long-term disruption of resistance determinants. In contrast, CRISPR-Cas12a (Cpf1) introduces staggered DSBs and possesses intrinsic RNase activity that enables self-processing of crRNA arrays, thereby facilitating efficient multiplex targeting (Ma et al., 2022; Swarts, 2019; Luo et al., 2025). Beyond targeted DNA cleavage, Cas12a exhibits collateral trans-cleavage activity against single-stranded DNA (ssDNA), which may contribute to destabilization of eDNA, a critical structural component of the biofilm matrix. Through simultaneous targeting of structural (*ica*) and regulatory (*agr*) genes, Cas12a not only disrupts biofilm formation but also enhances bacterial susceptibility to antibiotics, representing a mechanistically synergistic alternative to Cas9 (Swarts, 2019; Luo et al., 2025; Khan and Sallard, 2023).

**Figure 1:**
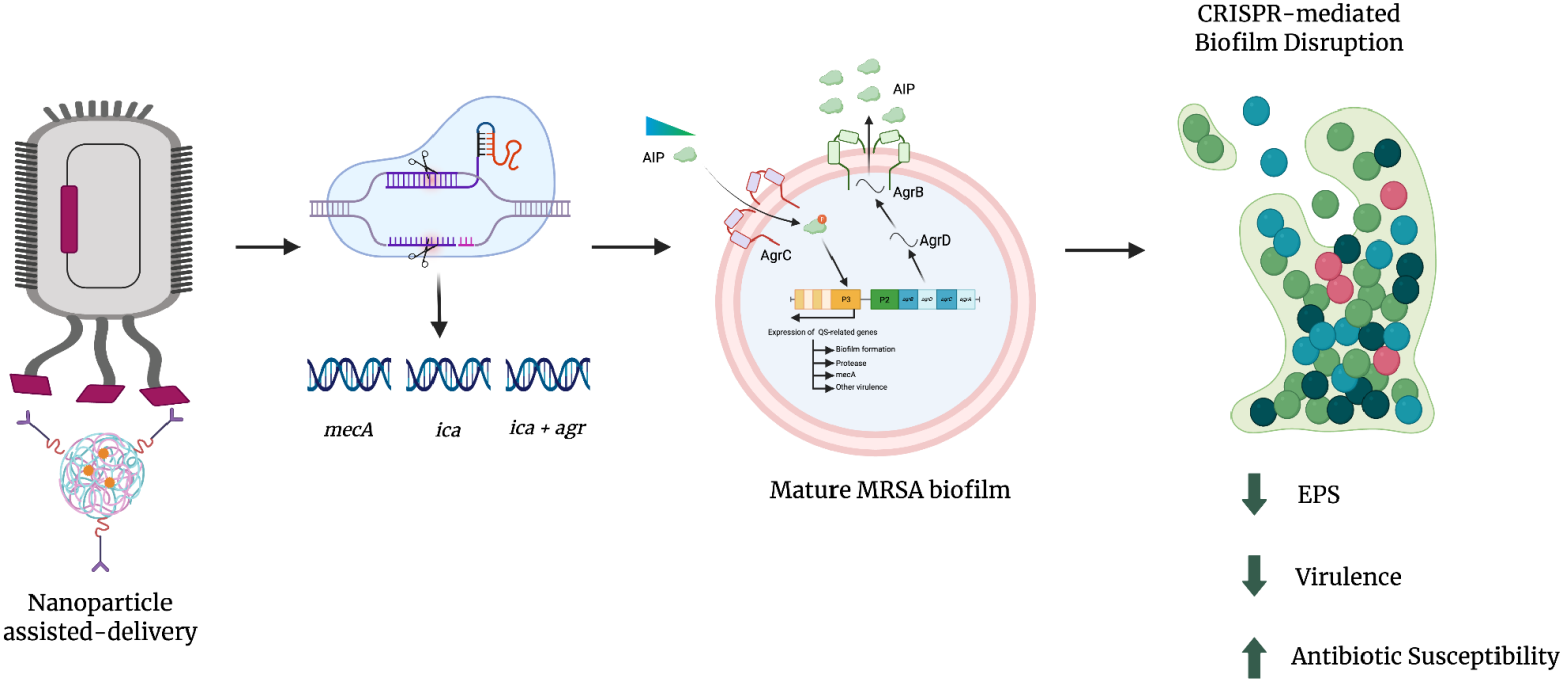
Mechanistic integration of CRISPR-Cas systems in the disruption of MRSA biofilm architecture.

CRISPR-Cas13 expands CRISPR functionality into the transcriptomic domain by targeting single-stranded RNA (ssRNA). Upon binding to complementary mRNA, Cas13 mediates RNA cleavage, effectively silencing gene expression without altering the genome (Zhang et al., 2024). In MRSA, targeting transcripts of *ica* and *agr* results in reduced synthesis of biofilm matrix components and quorum-sensing molecules. This post-transcriptional regulation offers a reversible and dynamic mechanism to suppress biofilm-associated phenotypes, making Cas13 particularly valuable for temporal control of virulence and as an adjunct to DNA-targeting systems. Cas9 and Cas12a facilitate durable disruption of resistance and virulence genes, while Cas13 enables dynamic and reversible suppression of gene expression, allowing precise temporal control of pathogenic traits. The integrated mechanisms by which CRISPR-Cas systems target resistance, biofilm formation, and regulatory pathways in MRSA are illustrated in **Figure 1**. Through coordinated targeting of key regulatory nodes such as *ica* and *agr*, these systems destabilize biofilm architecture, enhance antibiotic susceptibility, and attenuate virulence. As summarized in **Table 3**, the distinct mechanistic features of each CRISPR-Cas system, including differences in target molecules, cleavage patterns, collateral activity, and multiplexing capacity, underscore their complementary roles in designing precision antimicrobial strategies. This comparative framework highlights how the strategic combination of CRISPR platforms can overcome the limitations of single-modality approaches, offering a sophisticated and adaptable therapeutic paradigm for managing persistent MRSA biofilm-associated infections (De La Fuente-Núñez and Lu, 2017; Ong et al., 2025; Luo et al., 2025; Zhang et al., 2024).

**Table 3:** Comparative features of CRISPR-Cas systems in MRSA biofilm targeting.

Feature	Cas9	Cas12a (Cpf1)	Cas13	References
Target Molecule	DNA	DNA	RNA	Banerjee et al., 2024; De La Fuente-Núñez and Lu, 2017; Ma et al., 2022; Swarts, 2019; Zhang et al., 2024
Guide RNA	sgRNA (crRNA + tracrRNA fusion)	crRNA (self-processing)	crRNA	Banerjee et al., 2024; De La Fuente-Núñez and Lu, 2017; Swarts, 2019; Luo et al., 2025; Zhang et al., 2024
Cleavage Type	Blunt double-strand breaks (DSBs)	Staggered DSBs	Single-stranded RNA cleavage	Banerjee et al., 2024; De La Fuente-Núñez and Lu, 2017; Ma et al., 2022; Zhang et al., 2024
PAM Requirement	Yes (NGG, GC-rich)	Yes (TTTV, T-rich)	None (PFS-dependent in some cases)	Swarts, 2019; Ma et al., 2022; Zhang et al., 2024
Collateral Activity	Absent	Present (ssDNA)	Present (ssRNA)	Swarts, 2019; Luo et al., 2025; Zhang et al., 2024
Repair Mechanism	NHEJ / HDR	NHEJ / HDR	Not applicable	Banerjee et al., 2024; De La Fuente-Núñez and Lu, 2017
Multiplexing Capability	Limited	High (intrinsic crRNA processing)	Moderate	Swarts, 2019; Luo et al., 2025; Zhang et al., 2024
Primary Mode of Action in MRSA	Gene knockout (e.g., *ica*, *agr*)	Gene knockout + matrix destabilization	Gene silencing (post-transcriptional)	De La Fuente-Núñez and Lu, 2017; Ong et al., 2025; Luo et al., 2025; Zhang et al., 2024
Effect on Biofilm	Disrupts structure and virulence pathways	Enhances biofilm destabilization and antibiotic susceptibility	Reduces expression of biofilm and QS-related genes	De La Fuente-Núñez and Lu, 2017; Ong et al., 2025; Luo et al., 2025; Zhang et al., 2024
Therapeutic Advantage	Stable, heritable genome editing	Efficient multi-gene targeting and dual activity	Reversible, tunable gene suppression	Banerjee et al., 2024; Swarts, 2019; Zhang et al., 2024
Limitations	PAM restriction, off-target risks	Collateral damage, delivery complexity	Transient effect, RNA instability	Banerjee et al., 2024; Swarts, 2019; Luo et al., 2025; Zhang et al., 2024

## 6 *In vivo* validation of CRISPR-based strategies for MRSA biofilm control

*In vivo* models provide critical validation of CRISPR-based antibiofilm strategies by demonstrating therapeutic efficacy under physiologically relevant conditions. Preclinical studies using CRISPR-Cas9–engineered bacteriophages represent one of the most compelling approaches, combining intrinsic phage lytic activity with sequence-specific genome targeting. In rat models of MRSA-associated soft tissue infection and osteomyelitis, these engineered phages achieved significant reductions in bacterial burden and biofilm integrity compared with unmodified phage controls, with near-complete resolution observed in treated lesions (Hamilton et al., 2019; Wang et al., 2024). The therapeutic effect was further enhanced through localized delivery strategies, such as encapsulation within alginate hydrogels, which improved retention at infection sites and enabled sustained CRISPR exposure within biofilm matrices, resulting in superior bacterial clearance and reduced histopathological evidence of infection (Wang et al., 2024).

Beyond bacteriophage-mediated delivery, alternative *in vivo* CRISPR platforms have demonstrated promising results. Engineered mobile genetic elements, including staphylococcal pathogenicity islands (SaPIs), have been adapted to deliver CRISPR-Cas9 constructs targeting virulence determinants, effectively preventing abscess formation and improving survival in murine infection models (Ram et al., 2018). Similarly, implant-associated and device-related infection models have shown that CRISPR delivery via sustained-release systems, such as hydrogels and biofilm-responsive scaffolds, enhances bacterial clearance from colonized surfaces compared with conventional antibiotic therapy alone (Wang et al., 2024). Complementary model systems, including *Galleria mellonella* larvae and murine catheter-associated infections, further demonstrate the ability of CRISPR-based interventions to reduce bacterial load, disrupt biofilm stability, and impair quorum-sensing–regulated virulence pathways (You et al., 2023; Meeker et al., 2016; Bikard et al., 2014; Cobb et al., 2019).

Despite these encouraging findings, translational challenges remain substantial. Efficient delivery into dense and heterogeneous biofilm matrices continues to be a primary limitation, as the EPS restricts diffusion and cellular uptake of CRISPR constructs. Nanoparticle-based carriers and bacteriophage systems have shown promise in enhancing stability, targeting specificity, and intracellular delivery efficiency (Saffari-Natanzi et al., 2025; Hwang and Kim, 2025). However, concerns regarding off-target genome editing, host immune responses, and unintended horizontal gene transfer necessitate rigorous evaluation of safety and specificity (Muteeb et al., 2023; Rahman et al., 2025). Furthermore, variability in therapeutic outcomes across infection models, particularly between superficial and deep-seated infections, underscores the need to optimize delivery strategies and target selection. Collectively, current *in vivo* evidence supports the therapeutic potential of CRISPR-based antibiofilm strategies while highlighting the need for continued investigation to enable consistent and clinically translatable outcomes.

## 7 Delivery systems for CRISPR anti-biofilm therapy

### 7.1 Engineered bacteriophages

Bacteriophages have emerged as one of the most promising delivery platforms for CRISPR-based antimicrobials due to their inherent specificity for bacterial hosts and their ability to self-amplify at infection sites (Balcha and Neja, 2023). Clinical translation of phage therapy is already underway, as demonstrated by studies showing that intravenous administration of Myoviridae phage cocktails (e.g., AB-SA01) significantly reduces *S. aureus* burden without adverse effects (Kebriaei et al., 2020). Regulatory momentum has further accelerated this field, with the U.S. Food and Drug Administration approving phase I/II clinical trials in 2019 for phage therapy targeting ventricular-assist devices–associated infections (Voelker, 2019). Genetic engineering has expanded the therapeutic scope of bacteriophages by enabling delivery of CRISPR payloads directly into MRSA cells. For example, bacteriophage ΦNM1 has been used to deliver a CRISPR-Cas9 phagemid targeting *aph-3* and *mecA* in multidrug-resistant *S. aureus*, restoring susceptibility to kanamycin, methicillin, and tetracycline while suppressing bacterial proliferation (Khambhati et al., 2022; Bikard et al., 2014; Katayama et al., 2000). Similarly, an engineered M13 phage expressing Cas13a targeting *mecA* in *S. aureus* USA300 demonstrated enhanced bactericidal activity compared with Cas9-based constructs, highlighting effector-dependent differences in antimicrobial potency (Kiga et al., 2020). *In vivo* relevance has also been demonstrated using CRISPR-Cas9–modified temperate phages in rat models of osteomyelitis and soft tissue infection, where targeting of the *nuc* gene resulted in superior antibiofilm activity compared with conventional antibiotics. Notably, combinatorial delivery with fosfomycin via alginate hydrogels further enhanced sustained antibiofilm efficacy (Cobb et al., 2019). Despite these advances, translational barriers remain. Phage host-range specificity limits broad applicability, and the potential for generalized transduction of virulence or resistance genes raises biosafety concerns (Youssef et al., 2025). Nevertheless, advances in synthetic biology, including host-range engineering, deletion of transduction-associated genes, and precision genome editing, are actively addressing these limitations and positioning CRISPR-phage systems for expanded clinical evaluation (Khambhati et al., 2022).

### 7.2 Conjugative CRISPR systems

Conjugative CRISPR delivery exploits bacterial conjugation, a natural mechanism of horizontal gene transfer, to disseminate CRISPR-Cas modules across bacterial populations. This approach is particularly well suited to biofilms, where dense cellular organization and sustained cell-to-cell contact enhance plasmid transfer frequency. Conjugative plasmids encode a type IV secretion system and an origin of transfer (*oriT*), enabling efficient mobilization of large genetic payloads between bacterial cells (Waksman, 2019). Engineered conjugative plasmids carrying CRISPR–Cas9 or CRISPRi modules have been developed to propagate through pathogenic populations, effectively converting resistant bacteria into vectors for CRISPR dissemination (Bikard et al., 2014; Jain et al., 2022). *In vitro* studies demonstrate that these systems can selectively eliminate antibiotic resistance plasmids, silence essential genes, and resensitize bacterial communities to conventional antibiotics (Neil et al., 2021). One particularly impactful application is CRISPR-mediated plasmid curing, in which Cas9 or Cas3 targets replication or resistance loci on plasmids encoding β-lactamase or *vanA*, leading to plasmid destabilization and loss (Rodrigues et al., 2019; Neil et al., 2021; Citorik et al., 2014). Biofilms provide an optimal ecological niche for conjugative delivery, as horizontal gene transfer is intrinsically enhanced within structured microbial communities (Stalder and Top, 2016). However, this strategy faces several limitations, including spatial heterogeneity within biofilms, competition with resident plasmids, plasmid incompatibility, and the emergence of CRISPR resistance mechanisms (Mayorga-Ramos et al., 2023; Rodrigues et al., 2019). These challenges necessitate careful vector engineering and ecological consideration to ensure efficient and stable dissemination in complex microbial environments.

### 7.3 Localized delivery approaches

Localized delivery systems provide an alternative strategy for enhancing CRISPR efficacy in biofilm-associated infections by concentrating therapeutic agents at the site of infection while minimizing systemic exposure. Hydrogels represent a versatile class of delivery matrices due to their biocompatibility, tunable mechanical properties, and capacity to encapsulate CRISPR components such as plasmids or ribonucleoprotein complexes (Li and Mooney, 2016). These systems protect CRISPR payloads from degradation and enable controlled release through diffusion or biofilm-responsive triggers, including pH shifts and enzymatic activity (Carpa et al., 2022). Composite delivery platforms that integrate hydrogels with nanoparticles further improve stability, penetration, and retention of CRISPR constructs within dense biofilms, enhancing local therapeutic efficacy (Yoon et al., 2025). Advanced hydrogel dressings designed for chronic wound environments can also incorporate antimicrobial peptides or antibiotics, enabling synergistic disruption of biofilms while facilitating CRISPR uptake (Jia et al., 2023).

For implant-associated infections, CRISPR-infused surface coatings provide a targeted strategy to prevent or disrupt biofilm formation. Polymeric films, nanofiber scaffolds, and layer-by-layer assemblies have been engineered to release CRISPR agents in response to bacterial contact or environmental cues, enabling sustained, site-specific antimicrobial activity (Scalia and Najmi, 2025; Pursey et al., 2018; Gomaa et al., 2014; Campoccia et al., 2013; Rostami et al., 2024; Koo et al., 2017). Smart materials responsive to microbial enzymes or acidic microenvironments further refine spatial precision by triggering CRISPR release only upon biofilm establishment (Qiu and Park, 2001; Hoffman, 2012). Sustained-release kinetics are particularly important for targeting slow-growing or dormant biofilm subpopulations protected by the EPS matrix. By optimizing polymer composition, crosslink density, and stimulus-responsive elements, localized delivery systems can prolong CRISPR exposure and enhance gene-editing efficiency within mature biofilms (Pursey et al., 2018; Rostami et al., 2024; Flemming et al., 2016). Despite these advances, challenges remain in ensuring uniform distribution, long-term stability, and scalability for clinical application.

## 8 Challenges and translational barriers

Despite rapid advances in CRISPR-based antimicrobial technologies, several biological and technical barriers continue to limit their clinical translation for MRSA biofilm-associated infections. Efficient delivery of CRISPR components into biofilm-embedded bacteria remains a primary challenge, as the dense EPS matrix restricts diffusion and access to deeper bacterial populations. Although delivery platforms such as viral vectors, lipid nanoparticles, and polymeric carriers have been developed to enhance stability and cellular uptake (Seijas et al., 2025; Lee et al., 2025), their effectiveness is often constrained by physicochemical limitations, including aggregation of Cas proteins that impairs intracellular trafficking (Seijas et al., 2025). These challenges highlight the need for delivery systems specifically optimized for the heterogeneous and structured biofilm environment.

In addition to delivery constraints, issues of specificity, biosafety, and host interaction remain critical. Off-target effects resulting from guide RNA mismatch tolerance can lead to unintended genetic modifications, raising concerns about therapeutic reliability and safety, despite advances in high-fidelity Cas variants and computational design tools (Lotfi et al., 2025; Xu et al., 2026). Furthermore, CRISPR activity has been associated with large-scale genomic alterations, including chromosomal rearrangements that are difficult to detect using standard methods (Hunt et al., 2023). Host immune responses also pose a significant limitation, as Cas nucleases may trigger both innate and adaptive immunity, reducing efficacy and complicating repeat dosing (Li et al., 2018; Nguyen and Quang, 2025). The presence of pre-existing immunity further underscores the need for improved biocompatibility and immune-evasive delivery strategies.

Finally, resistance evolution, scalability, and regulatory considerations represent major hurdles to clinical implementation. Bacterial resistance to both CRISPR systems and their delivery vehicles, through mechanisms such as target-site mutation, acquisition of anti-CRISPR proteins, and phage resistance, can compromise long-term efficacy (Kadkhoda et al., 2024; Saeed et al., 2025; Ates et al., 2024). At the same time, the genomic plasticity of MRSA, driven by mutation, recombination, and horizontal gene transfer, facilitates rapid adaptation under selective pressure (Panneerselvam et al., 2025; Chen et al., 2020). Beyond biological challenges, large-scale manufacturing, regulatory approval, and long-term biosafety evaluation remain unresolved, requiring standardized frameworks to ensure safety, reproducibility, and ethical oversight (Ahmed et al., 2024; Du et al., 2025). Collectively, these limitations emphasize that while CRISPR-based strategies hold significant promise, their successful translation will depend on integrated advances in delivery design, targeting precision, and regulatory development. A structured overview of these barriers, including their mechanistic basis and current mitigation strategies, is presented in **Table 4**.

**Table 4:** Key challenges limiting clinical translation of CRISPR-based anti-biofilm therapies.

Challenge category	Underlying mechanism	Impact on CRISPR efficacy	Current mitigation strategies	Remaining gaps / Implications	References
Delivery Efficiency	Inefficient transport of CRISPR components into target bacterial cells, particularly within dense biofilm matrices	Reduced intracellular availability and diminished genome-editing efficiency	Nanocarriers and bacteriophage-based delivery platforms	Need for optimized delivery systems with enhanced targeting specificity, stability, and penetration within biofilms	(Ates et al., 2024)
Resistance Development	Emergence of bacterial resistance to CRISPR components or delivery vectors (e.g., phage resistance, target mutation)	Limits durability and long-term therapeutic effectiveness	Optimization of phagemid packaging to minimize wild-type phage contamination	Strategies to delay resistance evolution and sustain long-term efficacy remain underdeveloped	(Shimamori et al., 2024)
Specificity and Off-Target Effects	gRNA mismatch tolerance leading to unintended CRISPR activity	Risk of off-target genetic modifications affecting safety and therapeutic reliability	Use of CRISPRi and CRISPR-Cas13a for improved targeting precision	Further refinement of targeting accuracy and development of comprehensive off-target detection methods are required	(Guo et al., 2023)
Regulatory and Ethical Concerns	Absence of dedicated regulatory frameworks for CRISPR-based antimicrobials	Delays clinical translation and limits adoption in healthcare systems	Initiation of regulatory dialogue and development of ethical policy frameworks	Establishment of standardized regulatory guidelines and robust ethical oversight remains essential	(Gutiérrez et al., 2018)
Biofilm Penetration	Limited penetration of CRISPR systems through the EPS matrix	Reduced activity against biofilm-associated infections and heterogeneous bacterial populations	Combination with nanoparticles and phage-derived matrix-degrading enzymes	Development of biofilm-specific targeting and enhanced penetration strategies is still required	(Joshi et al., 2025; Ong et al., 2025)
Safety and Immunogenicity	Host immune recognition of CRISPR components and delivery carriers	Potential inflammatory responses and reduced efficacy upon repeated administration	Design of biocompatible and less immunogenic delivery systems	Long-term safety, immunogenicity, and repeat-dose feasibility remain poorly characterized	(Xu et al., 2025)
Cost and Scalability	High production costs and complex manufacturing processes for CRISPR systems and delivery vectors	Limits clinical accessibility and large-scale deployment	Integration with cost-efficient delivery platforms such as microneedles and nanocarriers (Davies, 2003)	Scalable, reproducible, and economically viable production pipelines remain necessary for widespread adoption (Davies, 2003)	(Shivpuje et al., 2025)

## 9 Future directions and emerging innovations

Future progress in CRISPR-based strategies against MRSA biofilms will depend on approaches that simultaneously address genetic complexity, biofilm resilience, and translational feasibility. Among emerging directions, self-targeting CRISPR systems represent a particularly promising strategy, enabling sequence-specific disruption of genes associated with antibiotic resistance, virulence, and persistence while maintaining programmability to adapt to evolving resistance profiles and clonal heterogeneity (Ma and Lu, 2025; Mayorga-Ramos et al., 2023). This adaptability is especially relevant in MRSA populations characterized by high genomic plasticity. In parallel, multiplexed CRISPR platforms have emerged as a critical advancement, allowing simultaneous targeting of multiple regulatory and structural pathways, including quorum sensing, stress-response systems, resistance determinants, and matrix biosynthesis, thereby overcoming functional redundancy that stabilizes biofilm architecture (Kumar et al., 2025; Saffari-Natanzi et al., 2025). Integration of multiplex targeting with advanced delivery systems, such as nanoparticle-assisted CRISPR transport, further enhances penetration into dense biofilm matrices and improves intracellular delivery efficiency (El-Sayed et al., 2025).

Advances in artificial intelligence (AI) are expected to further accelerate CRISPR optimization by improving guide RNA design and targeting precision. Machine learning–based approaches enable more accurate discrimination between on-target and off-target sequences, reducing unintended genomic alterations while enhancing editing efficiency (Kim et al., 2025; Liberty et al., 2025; Saxena et al., 2024). When combined with high-fidelity Cas variants, these strategies provide a pathway toward safer and more reliable CRISPR-based therapeutics. Concurrently, the convergence of CRISPR technologies with bacteriophage-based delivery systems offers a biologically adaptive platform for targeting MRSA within biofilms. Phage-mediated CRISPR delivery leverages host specificity and self-amplification, enabling localized and efficient genome editing, while engineered phage cocktails mitigate resistance development by expanding host range and distributing selective pressure across multiple targets (He and Chen, 2024; Rahman et al., 2025). These approaches collectively reflect a shift toward precision antimicrobial strategies that minimize disruption of commensal microbiota while maintaining high specificity against pathogenic populations.

Beyond therapeutic applications, CRISPR-based theranostic platforms represent an emerging paradigm that integrates rapid detection with targeted intervention. Cas12- and Cas13-based systems enable sequence-specific identification of MRSA while simultaneously modulating resistance and virulence-associated pathways, providing a rapid-response framework for managing severe or drug-resistant infections (Nouri et al., 2025). Cas13-mediated RNA targeting offers a reversible, non-genomic approach to suppress virulence networks, potentially reducing selective pressure and improving biosafety profiles (Wang et al., 2025). Despite these advances, significant barriers, including delivery optimization, off-target effects, resistance evolution, and regulatory constraints, must be addressed to enable clinical translation. Ultimately, progress in this field will depend on the coordinated integration of CRISPR engineering, smart delivery systems, computational design tools, and scalable manufacturing pipelines, supported by rigorous preclinical validation and regulatory standardization. Such a systems-level approach is essential to translate CRISPR technologies into clinically viable solutions for the effective control of persistent MRSA biofilm-associated infections (Glass et al., 2018).

## 10 Conclusion

MRSA biofilms remain difficult to treat due to their structural and physiological complexity, which limits the effectiveness of conventional antimicrobial therapies. While CRISPR-Cas systems provide a targeted approach to disrupt resistance and biofilm-associated pathways, their application in these systems is still in the early stages. This review highlights the importance of aligning CRISPR-based strategies with the biological characteristics of MRSA biofilms, rather than focusing solely on gene-level targeting. Although emerging studies, including *in vivo* models, demonstrate promising outcomes, significant challenges in delivery, specificity, and safety must be addressed. Overall, CRISPR represents a potential tool for improving the management of MRSA biofilm-associated infections, but further optimization and validation are required before clinical application.
